# Covering All the Bases: Complementary MR1 Antigen Presentation Pathways Sample Diverse Antigens and Intracellular Compartments

**DOI:** 10.3389/fimmu.2020.02034

**Published:** 2020-09-02

**Authors:** Corinna Kulicke, Elham Karamooz, David Lewinsohn, Melanie Harriff

**Affiliations:** ^1^Pulmonary and Critical Care Medicine, Oregon Health and Science University, Portland, OR, United States; ^2^VA Portland Health Care System, Research and Development, Portland, OR, United States; ^3^Department of Pediatrics, Oregon Health and Science University, Portland, OR, United States; ^4^Department of Molecular and Microbial Immunology, Oregon Health and Science University, Portland, OR, United States

**Keywords:** antigen presentation, MR1, MAIT cell, ligands, endosomal trafficking

## Abstract

The ubiquitously expressed, monomorphic MHC class Ib molecule MHC class I-related protein 1 (MR1) presents microbial metabolites to mucosal-associated invariant T (MAIT) cells. However, recent work demonstrates that both the ligands bound by MR1 and the T cells restricted by it are more diverse than originally thought. It is becoming increasingly clear that MR1 is capable of presenting a remarkable variety of both microbial and non-microbial small molecule antigens to a diverse group of MR1-restricted T cells (MR1Ts) and that the antigen presentation pathway differs between exogenously delivered antigen and intracellular microbial infection. These distinct antigen presentation pathways suggest that MR1 shares features of both MHC class I and MHC class II antigen presentation, enabling it to sample diverse intracellular compartments and capture antigen of both intracellular and extracellular origin. Here, we review recent developments and new insights into the cellular mechanisms of MR1-dependent antigen presentation with a focus on microbial MR1T cell antigens.

## Introduction

The immune system is traditionally thought of as dichotomous. On the one hand, the innate immune system is activated by broadly conserved pathogen-associated molecular patterns (PAMPs) detected by germline-encoded pattern recognition receptors. By contrast, the adaptive immune response relies on somatic re-arrangement of antigen receptor genes to generate the diversity and specificity needed to sense extensively processed peptide antigens in the context of highly polymorphic major histocompatibility complex (MHC) molecules. However, it is increasingly appreciated that these categories rather represent the extremes of a spectrum with non-classical immune cell subsets such as innate-like, donor-unrestricted T cells (DURTs) defying a binary classification (1–3). Instead, DURTs have attributes of both adaptive and innate immunity. For example, while they express somatically re-arranged T cell receptors (TCRs), their TCR repertoire is limited, and in many cases semi-invariant. Moreover, although these TCRs recognize their cognate antigen in the context of antigen presenting molecules, they are restricted by highly conserved, monomorphic proteins displaying primarily non-peptidic ligands (1, 3). One of these is the MHC class Ib molecule MHC I-related protein 1 (MR1). First identified in 1995 as an MHC I-related gene encoded outside the MHC locus (4), MR1 was later found to be the restricting element of the innate-like mucosal-associated invariant T (MAIT) cells (5). Like other non-conventional T cell subsets, these cells express a limited TCR repertoire and rapidly exert effector functions upon activation [recently reviewed in (6)]. While classical MAIT cells are defined by expression of the TRAV1-2 TCRα chain, more recent work has identified TRAV1-2^−^ T cells that are activated in an MR1-dependent manner, expanding the family of MR1-restricted T cells (MR1Ts) (5, 7–11). The first MAIT cell-activating MR1 ligands identified were intermediates produced during the microbial biosynthesis of riboflavin (vitamin B2) (12, 13). Since mammalian cells do not express the enzymes of this biosynthetic pathway, riboflavin precursors represent a microbe-derived molecular pattern (14). Ongoing ligand identification efforts have revealed many more microbial and non-microbial MR1 ligands which comprise both agonists and antagonists of MR1T cell activation (9, 15–17). Importantly, the number of microorganisms that synthesize riboflavin or other putative MR1 ligands is large and includes many commensal species in addition to pathogens (18, 19). This, together with the frequency of MR1Ts and the ubiquitous expression of MR1, likely necessitates tight regulation of MR1 antigen presentation to prevent inappropriate activation (20).

Classical peptide antigen presentation relies on a division of labor on the molecular scale: ER-resident MHC class I molecules bind and present peptides derived from intracellular protein synthesis whereas MHC class II molecules survey endosomal compartments where they encounter extracellular material taken up by endo- or phagocytosis. Although there are exceptions to this paradigm such as cross-presentation of exogenous or particulate antigen on MHC class I molecules, broadly speaking, immune surveillance of endogenous and exogenous peptide antigen is achieved by compartmentalization of two different antigen presenting molecules with distinct intracellular trafficking patterns (21). By contrast, MR1 is the only known metabolite-presenting molecule, placing the burden of sampling both intracellular and exogenous sources of antigenic metabolites on a single molecule. Accordingly, it is becoming increasingly clear that MR1T antigens are presented through multiple specialized presentation mechanisms likely reflecting the biochemical properties as well as the abundance and intracellular distribution of the antigen (20, 22).

In this review, we will discuss recent advances in our understanding of both the expanding repertoire of MR1 ligands and current models for distinct pathways by which these ligands are presented to MR1Ts.

## Toward Defining the MR1 Ligandome

### Canonical MAIT Cell Antigens: Lumazines, Pterins, and Pyrimidine Neoantigens

The identification of microbial riboflavin metabolism as a source of MAIT cell-activating ligands marked a major breakthrough for the MR1 field (12, 13). The first microbial MAIT cell antigens to be identified were ribityllumazine metabolites upstream of riboflavin (vitamin B2) biosynthesis. These MR1 ligands include 6,7-dimethyl-8-D-ribityllumazine (DMRL), 7-hydroxy-6-methyl-8-D-ribityllumazine (HMRL), and reduced 6-hydroxymethyl-8-D-ribityllumazine (rRL) (13). In the same report, Kjer-Nielsen et al., described the MAIT antagonist ligand 6-formylpterin (6-FP), which derives from folate (vitamin B9). 6-FP has the same bicyclic ring structure as the ribityllumazines but lacks the ribityl tail, which is critical for recognition by the MAIT TCR (23). A subsequent report described the formation of the pyrimidine neoantigens 5-(2-oxo-propylidenamino)6-D-ribitylaminouracil (5-OP-RU) and 5-(2-oxoethylideneamino)-6-D-ribitylaminouracil (5-OE-RU), which form upon the spontaneous reaction of the riboflavin precursor 5-aminoribityluracil (5-A-RU) with methylglyoxal or glyoxal, respectively (12). 5-OP-RU and 5-OE-RU have a single ring structure, but still feature the ribityl moiety. In a unique mode of antigen binding, both 6-FP and the pyrimidine neoantigens covalently bind to MR1 by forming a Schiff base with lysine residue 43 (K43) at the bottom of the MR1 antigen binding groove (12, 13). The ribityllumazine ligands, on the other hand, non-covalently associate with MR1, which correlates with lower antigenicity (12, 16, 24). More recently, we identified the additional MR1T-activating ribityllumazine ligands photolumazine I (PLI) and photolumazine III (PLIII), as well as the antagonistic ligand 7,8-didemethyl-8-hydroxy-5-deazariboflavin (FO) (15). All of these ligands could have 5-A-RU as a precursor metabolite, suggesting that it may be a key molecule in the synthesis of MR1T antigens. In support of this, modulation of riboflavin biosynthesis correlates with MR1T activation (25) and deletion of the enzyme responsible for the synthesis of 5-A-RU abrogates MR1T recognition of some microbes (12, 13, 25, 26). Microbial vitamin B metabolites comprise the most potent and well-characterized MAIT cell agonists to date. However, recent studies described below clearly demonstrate that a much broader range of small molecule metabolites can bind to MR1 and activate MR1Ts.

### Beyond Vitamin B Metabolites: Evidence for Additional MR1 Ligands

Aspects of both MR1 itself and the MR1T TCRs support the hypothesis that the MR1 ligand repertoire includes other classes of molecules in addition to those in the vitamin B family. MR1 is structurally similar to other MHC class I molecules in that its heavy chain consists of three extracellular domains (α_1_-α_3_), a transmembrane domain, and a small cytosolic tail. Like MHC class Ia, MR1 non-covalently associates with β_2_-microglobulin (β_2_m) to form a heterodimer (27). The MR1 antigen binding cleft is formed by the α1 and α2 domains of the heavy chain and consists of an A' and an F'-pocket (23, 24, 28, 29). The canonical antigens described above bind in the A'-pocket, which consists primarily of hydrophobic amino acids (13, 28). The non-polar nature of these residues accommodates organic ligands such as the vitamin B metabolites (30) and there are various other classes of small molecules with chemical properties consistent with binding in this groove (15, 16, 30). Furthermore, the unoccupied space remaining in the MR1 ligand binding groove outside of the A' pocket leaves open the possibility for binding of additional ligands or chaperones (31). Recent advances in defining the TCRs restricted by MR1 support this notion. MAIT cells were originally defined by their semi-invariant TCR, which consists of the TRAV1-2 α chain paired with a limited number of β chains, and features a signature CDR3α sequence (11). However, numerous studies continue to expand the MR1T TCR repertoire [reviewed in: (6, 32)]. While many of the MR1Ts with non-canonical MAIT TCRs recognize the vitamin B metabolite ligands, there are others that do not (9, 10, 33). Additionally, even among those TCRs that do recognize vitamin B metabolites, there is differential recognition of individual ligands by distinct TCRs (7, 15, 34). Combined with the conformational plasticity of the MR1 binding groove (13, 28), the increasingly recognized diversity in MR1-restricted TCRs suggests the repertoire of ligands is likely to be much broader than the vitamin B metabolites.

### New Classes of MR1 Ligands: Synthetic Compounds, Riboflavin-Deficient Bacteria, and Cancer Metabolism

Inspired by the reasoning presented above, ongoing ligand identification efforts have discovered a number of non-vitamin B-derived MR1 ligands, both microbial- and non-microbial. The first non-vitamin B-derived ligands were identified through *in silico* modeling of putative MR1 interactions with synthetic molecules in chemical compound libraries (16). These ligands include the synthetic drug compounds diclofenac, an aspirin analog (3-formylsalicylic acid), and a methotrexate derivative (2,4-diamino-6-formylpteridine). Like the ribityllumazines and pyrimidines, these drugs are small cyclic compounds, some of which are MAIT cell agonists and some of which are antagonists (16). The role these ligands may play in drug-induced immune modulation through MAIT cell activation or inhibition is not yet clear. Using a similar *in silico* screen, Salio et al., recently expanded the library of MR1 ligands, including the first molecule that prevents MR1 egress from the ER (17). Intriguingly, this ligand binds in the MR1 A'-pocket in a non-covalent fashion and prevents MR1 surface translocation and MAIT cell activation in response to canonical ligands (17).

In addition to these synthetic molecules, we have found evidence for the existence of non-vitamin B metabolite microbial MR1 ligands. For example, we identified a TRAV12-2^+^ MR1T clone that responds to an unidentified ligand from *Streptococcus pyogenes* (*S. pyogenes*), a bacterium that does not express the enzymes of the riboflavin biosynthetic pathway (10). We also performed mass spectrometry on MR1 molecules purified from cells infected with *Escherichia coli (E. coli)* or *Mycobacterium smegmatis* (*M. smegmatis*) (15). While the canonical ribityllumazine and pyrimidine ligands were the most common ions bound to the MR1 purified from *E. coli*-infected cells, these ligands had relatively low abundance in the molecules purified from *M. smegmatis*-infected cells. MR1 preparations from either infection contained ions with an ionization pattern not consistent with ribityllumazine molecules (15). Together, these data suggest a distinct class of small molecules metabolite ligands that is likely to be more prevalent in *M. smegmatis*. Consistent with this notion, Corbett et al., reported that some MR1 ligands were differentially abundant in *E. coli* compared to *Salmonella typhimurium* (*S. typhimurium*) (12). Together with data demonstrating the differential recognition of ligands by distinct MR1T TCRs (15), evidence that infection with different microbes drives the expansion of MR1Ts with distinct β chains (35) further supports the idea that different bacterial species express different ligands.

Moreover, a number of studies have recently provided indirect evidence for non-microbial self-ligands for MR1 (9, 15, 33). In the same study describing novel microbial MR1 ligands by mass spectrometry, we also identified numerous unique ions associated with MR1 purified from uninfected insect cells. We hypothesize that some of these ions represent novel endogenous MR1 ligands, whereas others may originate from chaperones or cellular co-factors associated with MR1 loading and trafficking (15). Others have demonstrated the existence of putative self-ligands in the context of tumor cell lines and primary cancers. For example, Lepore et al., identified a population of TRAV1-2^−^ MR1Ts that recognize molecules derived from tumor cells and not microbes or cell culture medium (9). Similarly, Crowther et al. generated a non-MAIT MR1T clone specifically responding to cancer cell lines and primary cancer cells (33). Interestingly, the tumor-associated antigens reported by Lepore et al. did not form a Schiff base with MR1 like the pyrimidines and the pterins but were more similar to the ribityllumazine ligands in their non-covalent interaction with the antigen presenting molecule. The chemical identity of these MR1T antigens remains to be determined. Crowther et al. hypothesized that the ligand recognized in their system was derived from the altered metabolism characteristic of neoplastic transformation but did not report its identity. Since all remain to be identified, it is still unknown whether any of these potential self-ligands are present in healthy cells and may play a role as chaperone-like molecules such as Ii, serve as MR1T antigens that contribute to inflammation, or constitute regulatory MR1 ligands involved in immune modulation and tolerance.

## Distinct and Complementary MR1 Antigen Presentation Pathways

### MR1 at Steady State: ER and Vesicular Pools but Not Much at the Cell Surface

**Figure 1:**
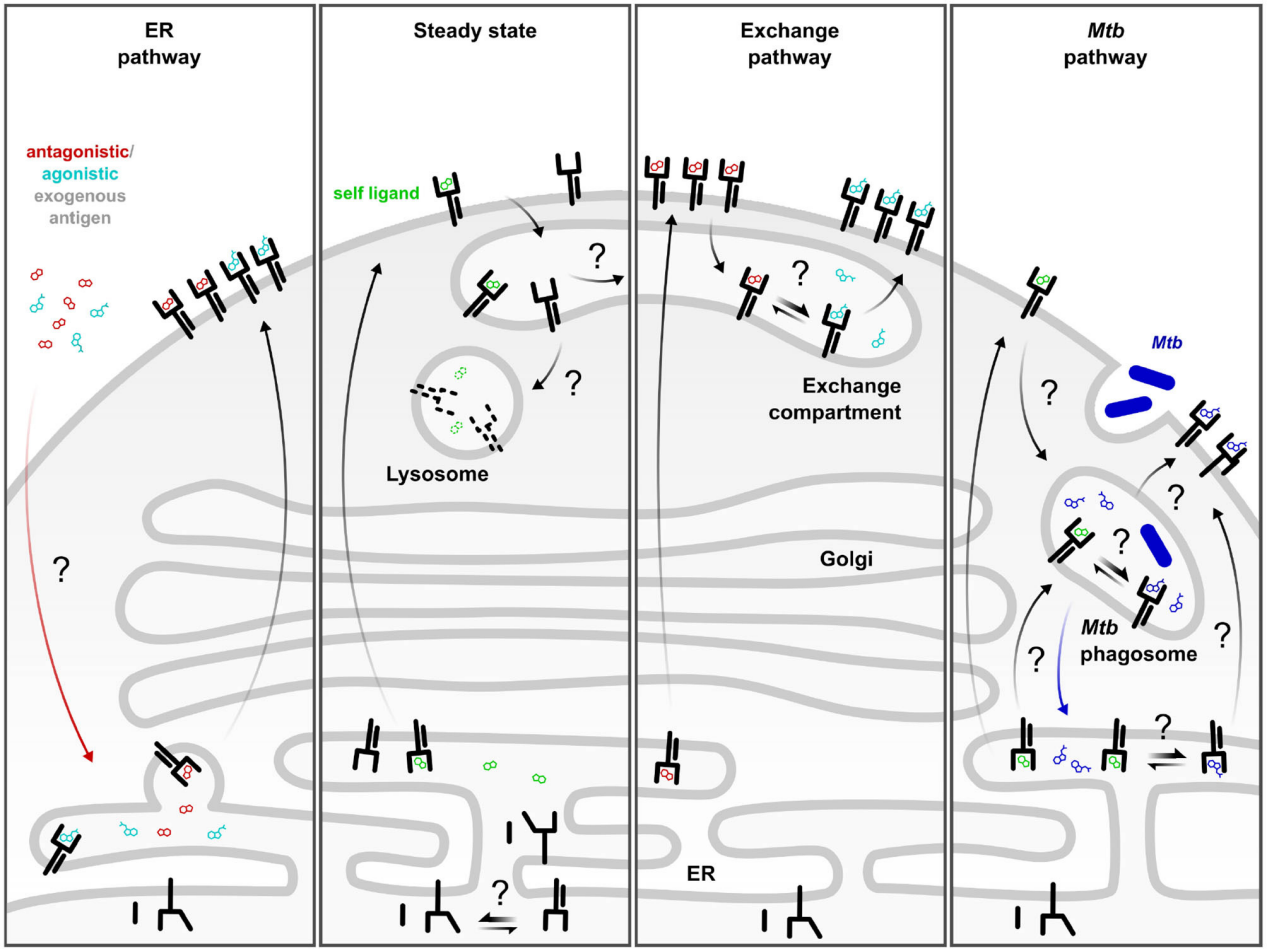
Complementary MR1 antigen presentation pathways. ER pathway: Defined antagonistic and agonistic exogenous MR1 ligands including 6-FP and 5-OP-RU access incompletely folded MR1 in the ER and trigger its translocation to the cell surface. This pathway is dependent on neutralization of the K43 residue. Steady state: At baseline, a small fraction of MR1 molecules constitutively leaves the ER, potentially as a result of the conformational equilibrium of MR1 or through binding to an unknown self-ligand or a ubiquitous environmental ligand. These molecules are rapidly internalized and degraded in the absence of exogenous ligand or microbial infection. Exchange pathway: Alternatively, the self-ligand may be exchanged for exogenous, antigenic ligand in an exchange compartment from where the re-loaded MR1 can return to the cell surface. This process can be amplified by TLR stimulation or by pre-incubation with MR1-stabilizing antagonist ligand which increases the pool of post-ER MR1 available for exchange. *Mtb* pathway: Presentation of mycobacterial MR1T antigens generated upon intracellular infection are likely loaded in a *Mycobacterium tuberculosis* (*Mtb*)-specific phagosomal compartment. MR1 molecules may be delivered to the *Mtb* phagosome directly or *via* internalization from the cell surface. Alternatively, *Mtb*-derived ligand may reach the ER and induce MR1 surface translocation. Black arrows denote movement of MR1. Colored arrows denote movement of ligand. Straight double arrows indicate equilibria. Question marks denote hypothetical steps which have not been defined mechanistically.

While MR1 has been consistently found to localize to the ER, it has also been reported to co-localize with late endosomal proteins (30, 36, 37). We have observed a vesicular distribution of MR1 even in the absence of exogenously provided ligands (36), indicating that constitutive egress from the ER is possible. However, since endogenous MR1 is hardly detectable at the cell surface of most cell lines and primary cells, these molecules are likely very transiently expressed at the cell surface and either sequestered in intracellular stores or rapidly degraded upon internalization (38) (Figure 1, “steady state”). This hypothesis is supported by the ability of an anti-MR1 antibody to stabilize transiently expressed MR1 molecules at the cell surface (39) and the observation that MR1 detection by flow cytometry is cumulative when cells are incubated with antibody under conditions that allow internalization of MR1-antibody complexes (40). Of note, microbe-induced upregulation of MR1 surface expression was independent of MR1 ligand in the same study. Instead, toll-like receptor (TLR) signaling increased MR1 surface levels in some but not all antigen presenting cells and both this modulation as well as steady state MR1 surface expression were dependent on NF-κB (40). Importantly, pre-treatment with a TLR2 agonist that induced upregulation of MR1 surface expression increased MR1-dependent antigen presentation in the same report (40), providing evidence that increased anterograde flux of MR1 could feed into an “exchange pathway” (Figure 1, discussed below). Furthermore, McWilliam et al. showed that incubation with 5-OP-RU led to the detection of a small number of MR1-5-OP-RU complexes even at 4°C, supporting the existence of loadable MR1 molecules at the cell surface at steady state (41). MR1 requires ligand binding for stable association with β_2_m and acquisition of EndoH resistance, a marker of ER egress (41). Therefore, we expect that any MR1 molecules in subcellular compartments other than the ER carry a ligand. The two most likely sources of this molecule are derivatives of folate and riboflavin contained in the culture medium or an endogenous self-ligand (discussed above). Alternatively, a small proportion of ER-resident MR1 molecules may stochastically acquire a conformation that allows them to leave the ER as a result of a conformational equilibrium, as proposed by McWilliam and Villadangos based on similar concepts in MHC class Ia folding (42–44). Regardless of how different MR1 molecules reach their respective intracellular locations, the existence of ER, vesicular, and cell surface pools of MR1 conceivably contribute to the sampling of different intracellular environments harboring different sources of MR1T antigens (20). This notion is not without precedent as different pools of MHC class Ia molecules similarly survey different subcellular compartments. Specifically, nascent MHC class Ia molecules present antigens loaded in the ER whereas a subset of recycling molecules is thought to be loaded with exogenous antigen in other compartments in the context of cross-presentation [reviewed in (45)]. Notably, microscopic localization studies of MR1 so far have relied on overexpression of GFP-tagged versions of the molecule (36, 41). It would be extremely informative to directly investigate the intracellular distribution of endogenous MR1 but this has so far been prevented by the prohibitively low abundance of the protein in WT cells.

### The ER Pathway: Surface Translocation in Response to Exogenous Ligand

The use of defined model antigens such as 5-OP-RU in both *in vitro* and *in vivo* models has enabled valuable insights into the mechanisms of MR1-mediated presentation of soluble ligands administered exogenously. These studies have elucidated an “on-demand” mode of presentation for MR1 in this context (38, 41). Here, MR1 resides primarily in the ER in a partially folded, ligand-receptive state. Only when exogenous ligand neutralizes the positive charge on the K43 residue in the MR1 binding groove can the protein associate with β_2_m and acquire the ability to leave the ER and translocate to the cell surface (38, 41) (Figure 1, “ER pathway”). MR1 loading in this model is thought to take place in the ER, although the mechanism by which the MR1 ligand reaches this compartment is still unclear (22, 38). This pathway has been the subject of excellent previous reviews to which we refer the reader [Example: (43)].

### The Exchange Pathway: Swapping Out Ligands on Recycling MR1 Molecules

Recent work by our group suggests that ligand exchange plays an important role in MR1-mediated presentation of exogenous antigen (31). In this study, pre-incubation of a bronchial epithelial cell line with 6-FP overnight enhanced the presentation of exogenous ligand but did not affect MR1T activation in response to *Mycobacterium tuberculosis* (*Mtb)* infection. This supports a model in which pre-incubation with MR1-stabilizing ligand brings MR1 to the cell surface and from there into an exchange compartment where it can be re-loaded with exogenous antigen [(20, 22); Figure 1, “exchange pathway”]. This model is consistent with work from McWilliam et al., who showed that re-loading of 6-FP-bound molecules was possible at 37°C but not on ice, indicating a requirement for internalization and recycling for ligand exchange to occur (41). Similarly, presentation of a set of novel MR1 ligands was reduced in cells over-expressing GPI-linked MR1 compared to those transduced with the WT protein, suggesting that a motif in the MR1 cytoplasmic tail may be required for ligand exchange and loading of some ligands (17). The notion of post-ER loading of MR1 molecules is further supported by the observation that pre-incubation with 6-FP rendered the subsequent surface expression of MR1-5-OP-RU complexes less sensitive to Brefeldin A (BFA) (41). Importantly, there was still a contribution of ER-derived molecules in this system as BFA partially reduced the MR1 surface levels (41).

Intriguingly, a shorter pre-incubation with 6-FP was previously shown to abrogate presentation of *M. smegmatis* supernatant (36), seemingly contradicting the hypothesis of an exchange pathway supplied with MR1 molecules that leave the ER bound to endogenous or exogenous antagonist ligands. These two observations could, however, be reconciled by a time-dependent model of MR1 trafficking. In this scenario, the majority of the MR1 molecules are occupied by 6-FP and localized to the cell surface after 2 h while overnight incubation allows enough time for internalization and recycling to the cell surface to occur. Consequently, ligand exchange is only observed upon the longer pre-incubation. McWilliam et al. reported much faster recycling kinetics for MR1, but these measurements were made in the hematopoietic cell line C1R (41) whereas our exchange studies were carried out in epithelial cells (31, 36). Thus, recycling kinetics might be different between cell lines, particularly since C1R cells are phagocytic professional antigen presenting cells whereas epithelial cells are not. Alternatively, the different outcomes following short compared to long 6-FP pre-incubation could be explained by the presence or absence of the antagonist during antigen presentation. Specifically, 6-FP was present for the duration of the ELISPOT after the short pre-incubation whereas the antagonist was washed off before co-culture with T cells after overnight incubation with 6-FP (31, 36). Thus, 6-FP was present during initial MR1 loading in both cases but was only present during presentation to MR1Ts in the 2 h pre-incubation experiment. While the rate of MR1 internalization from the cell surface is independent of ligand binding (41), the efficiency of ligand exchange or other aspects of MR1 antigen presentation could be differentially affected in the presence of the antagonist. Since exchange likely depends on the relative concentrations of the alternative ligands, the continuous presence of 6-FP in the first study may have shifted the equilibrium toward MR1 occupied by the antagonist.

### The Mtb Pathway: A Requirement for Intracellular Infection and Many Open Questions

The intracellular pathogen *Mtb* was one of the first to be discovered to produce MR1T antigens (18, 46). Nevertheless, how MR1 ligands are loaded in the context of intracellular microbial infection is less well defined compared to soluble, exogenous ligands. In fact, accumulating evidence demonstrates that the molecular machinery required for the presentation of microbe-derived antigen differs from that involved in the loading of exogenous ligand (20, 22, 38). For example, presentation of whole fixed *E. coli* bacteria is reduced upon inhibition of lysosomal acidification whereas MAIT cell activation by bacterial supernatant is not (40). In the same study, presentation of exogenous bacterial supernatant correlated with MR1 expression whereas presentation of MR1T antigen from intact bacteria was less dependent on MR1 over-expression (40). Similarly, we showed that Stx18 and VAMP4 both affect *Mtb* presentation but only Stx18 also affects surface translocation of MR1 in response to the stabilizing ligand 6-FP (36). This is consistent with early work by Huang et al., who showed that MR1 surface levels do not necessarily correlate with the ability to activate MAIT cells and that these two read-outs have different requirements for endosomal trafficking (47). Of note, these early experiments were carried out in the absence of bacterial infection or defined MR1T antigen.

Using transwell assays, we demonstrated that intracellular infection of the antigen presenting cell was required for the activation of MR1Ts in response to *Mtb* (48). By contrast, the supernatants of other bacteria such as *E. coli, M. smegmatis*, and *S. pyogenes* contain MR1 ligands capable of activating MR1Ts without the need for bacterial infection (10, 31, 36, 40). This difference could be explained by a comparatively low abundance of MR1 ligands produced by *Mtb*. As a consequence, containment of the microbe in an endosomal compartment might be necessary to achieve sufficiently high local concentrations of the antigen for MR1 loading. As mentioned above, gene expression levels of key enzymes of riboflavin synthesis correlated with the extent of MAIT cell activation in different *Streptococcus pneumoniae* isolates (25). Thus, it is plausible that such differences exist at the species level also. Alternatively, *Mtb* may not produce secreted MR1 ligands and liberation of MR1T antigens may require endosomal processing (31). Supporting a role for intracellular infection for optimal MR1 presentation of other intracellular pathogens, Le Bourhis et al. showed that rendering *Shigella flexneri* incapable of invading HeLa cells drastically reduced its ability to activate MAIT cells (49). Similarly, a *S. typhimurium* mutant unable to actively invade non-phagocytic cells did not elicit a MAIT cell response *in vivo* (50). However, supernatant from this mutant still activated an MR1-restricted Jurkat T cell clone *in vitro*, highlighting different mechanistic requirements for *in vitro* presentation of exogenous antigens compared to microbial infection under physiological conditions (50). Interestingly, administration of 5-OP-RU alone was not sufficient to induce MAIT cell accumulation in murine lungs although MAIT cells were activated as measured by CD69 expression (51). Accumulation of MAIT cells in this model was dependent on TLR signaling and could be achieved by co-administration of 5-OP-RU with a riboflavin-deficient bacterium or purified TLR ligands (51). This may either highlight a need for bacterial infection for optimal MR1 antigen presentation *in vivo* or indicate that MAIT cell expansion requires TLR-induced cytokine production at the site of infection. In another *in vitro* study, addition of fixed bacteria incapable of producing riboflavin did not increase MAIT cell activation in response to exogenously applied *E. coli* supernatant, indicating that both bacterium and ligand have to be present in the same compartment for optimal presentation in the context of MR1 in this model (40). Overall, the requirement for intracellular infection as well as the molecular mechanisms employed for antigen loading and presentation likely depend on the specific features of the infecting microbe. The metabolic state of the bacterium, the identity and stability of the MR1 ligands it produces, and the biochemical conditions it encounters in the intracellular environment are all likely determinants of the cellular mechanisms required for efficient MR1-mediated antigen presentation.

As mentioned, the requirement for intracellular infection with *Mtb* may indicate that endosomal processing is needed to generate and/or load mycobacterial antigen. While the cell surface can be a source of antigen presenting molecules that are loaded in endosomal compartments (45, 52), other theoretical possibilities include direct recruitment of MR1 molecules to the *Mtb* phagosome or delivery of *Mtb*-derived MR1 ligands to the ER (Figure 1, “Mtb pathway”). Indeed, both classical and non-classical MHC class I molecules have been detected in purified *Mtb* phagosomes (53). One way to account for the presence of these and other ER-resident proteins in phagosomes is the fusion of ER and phagosomal membranes (54, 55). Correspondingly, early evidence suggested that the ER endomembrane could contribute to phagosome membranes (54) although this has remained a point of contention [reviewed in (55)]. More recently, membrane contact sites (MCS), defined as points of close physical proximity between organelles which allow the exchange of lipids and ions without membrane fusion, have emerged as a potential explanation for the detection of ER material in phagosomal preparations (55). Alternative explanations for the presence of a subset of ER proteins in phagosomes include ER-to-phagosome vesicular trafficking and delivery of MHC-I from recycling endosomes (45, 55, 56). Both have been extensively studied in the context of MHC class I-mediated cross-presentation and although many details remain to be elucidated, multiple studies have implicated the ER SNARE Sec22b and its interaction partner Stx4 in the delivery of ER proteins directly to endosomal compartments (45, 57, 58). In our hands, knock down of Sec22b resulted in reduced presentation of *Mtb*-derived MR1T antigens (36) whereas Stx4 knock down specifically affected the presentation of *M. smegmatis* supernatant without inhibiting responses to *Mtb* infection (31). Thus, the extent of mechanistic overlap between MHC class I cross-presentation and MR1-mediated antigen presentation remains to be determined. In fact, MR1 also associates with MHC class II chaperones under certain circumstances (47), although it is not dependent on these as evident from the observation that epithelial cells, which do not express MHC class II machinery, can present MR1T antigens (20, 36, 48). While the MR1 antigen presentation pathway(s) may intersect with both MHC class I and class II pathways, we expect that specialized machinery exists to allow for the juxtaposition of MR1 and microbe-containing compartments and look forward to their identification.

By contrast, it is more difficult to envision a scenario in which mycobacterial antigens or even entire microbes should gain access to the ER. Although recent work by Legoux et al. implies that 5-OP-RU can not only rapidly cross lipid bilayers but even traverse skin and organs to reach the thymus when topically applied to mouse ears (59), the mechanism of transport remains to be identified. We hypothesize that dedicated molecular machinery is in place to capture, stabilize, and shuttle MR1 ligands between organelles and, potentially, across longer distances on micro- and macro-anatomical scales. The identification of these chaperones for MR1 ligands is of high priority for the MR1T field.

## Outstanding Questions

Taken together, the current literature supports a model in which redundant and complementary pathways allow MR1 to sample discrete antigens from a variety of subcellular compartments [(20, 22, 38); Figure 1]. The chemical nature of the antigens may be critical to understanding these pathways, as different classes of ligands may be generated and presented through different pathways. The existence of previously identified neoantigens (e.g., 5-OP-RU), the observation of clusters of unidentified ligands that may represent novel neoantigens, and the evidence for self-ligands, demonstrate a need to continue working toward defining the MR1 ligandome. While there is overwhelming evidence that 5-OP-RU is a potent ligand for MR1Ts, antigens of the highest potency may not necessarily be those that are the most protective. The identification of additional ligands would also provide better tools to investigate how different sources and types of MR1T antigens relate to differential TCR recognition and clonal expansion. As such, a key outstanding question is whether and how ligand diversity contributes to memory formation. Finally, a pragmatic question regarding ligand diversity is whether ligands can be modified in order to improve stability, biosynthetic capability, bioavailability, deliverability, and other features that will be requirements if MR1Ts are to be targeted for vaccine or therapeutic development. In this respect, a number of groups have recently generated new synthetic versions of the known ligands, including glyco-analogs (60), monodeoxyribityl and monohydroxyalkyl analogs (61), and pro-drug analogs (62). A better understanding of ligand diversity will be required as work to modify ligands for therapeutic purposes moves forward.

The outstanding questions regarding MR1-mediated antigen presentation primarily center on the intracellular trafficking of both the antigen presenting molecule and its ligands. Firstly, it remains puzzling why endogenous surface levels of MR1 are extremely low, yet surface expression readily increases upon over-expression of the molecule even if cells are cultured in medium devoid of folate (41). This would indicate that ligand availability is not the only limiting factor and implicates MR1 protein abundance, too. It is tempting to speculate that there might be an active retention mechanism at play (20), similar to the extensive quality control governing the release of loaded MHC class I molecules (63, 64). Indeed, although it has been shown that neutralization of K43 in the MR1 ligand binding groove facilitates ER egress (41), how this is detected on a molecular level is not known. Interestingly, MR1 seems to be able to breach cellular quality control mechanisms and translocate to the cell surface in a fully folded state upon incubation at 26°C (65). This is consistent with the idea that the molecular feature, likely a specific conformation, that releases MR1 from the ER, can be achieved without addition of exogenous ligand. We hypothesize that a key determinant of MR1 surface translocation is the extent of conformational plasticity in the heavy chain, as has been postulated in the context of peptide antigen presentation (63, 66, 67). Neutralization of K43 may be one of multiple ways to restrict conformational flexibility in a way that enables ER egress. Related questions pertain to the stability of partially folded MR1 in the ER and how stabilizing ligands reach this compartment. Moreover, the increasing evidence for multiple presentation pathways, including the notion of ligand exchange in endosomal compartments, opens the door to numerous questions concerning the molecular mechanisms governing exchange of MR1 ligands. Since Schiff bases are more labile in acidic environments (41), one possibility is that exchange occurs simply when internalized MR1 molecules reach a point in the endocytic pathway where the pH is sufficiently low to destabilize the covalent bond between MR1 and its ligand. As a result, the original ligand is released, and a new ligand can be bound to “empty” MR1 molecules. In this scenario, the equilibrium between MR1 molecules bound to each ligand is determined by the pH of the exchange compartment and the relative concentrations of the available ligands (Figure 1, “exchange pathway”). Alternatively, exchange could be an active process catalyzed by dedicated exchange chaperones, which have been described for other MHC molecules. Examples include TAPBPR for MHC class I (64), HLA-DM for MHC class II (68), and lipid transfer proteins for CD1 molecules (69). Taken together, it is becoming clear that different MR1 antigen presentation pathways enable the MR1-MR1T axis to sample various intracellular compartments while avoiding inappropriate MR1T cell activation. The relative contributions of these complementary pathways to protective MR1T cell immunity as well as the molecular machinery underlying the individual mechanisms remain to be established.

## Author Contributions

MH and CK wrote and edited the manuscript. CK generated the figure. EK and DL provided intellectual contribution to the topics covered and edited the manuscript. All authors contributed to the article and approved the submitted version.

## Conflict of Interest

The authors declare that the research was conducted in the absence of any commercial or financial relationships that could be construed as a potential conflict of interest. The handling editor declared a past co-authorship with one of the authors DL.
